# “How to Recognize if Your Child Is Seriously Ill” During COVID-19 Lockdown: An Evaluation of Parents' Confidence and Health-Seeking Behaviors

**DOI:** 10.3389/fped.2020.580323

**Published:** 2020-11-17

**Authors:** Emma Lim, Ravi D. Mistry, Alexandra Battersby, Kerry Dockerty, Aaron Koshy, Michelle N. Chopra, Matthew C. Carey, Jos M. Latour

**Affiliations:** ^1^Great North Children's Hospital, Newcastle upon Tyne, United Kingdom; ^2^Population Health Science Institute, Newcastle University, Newcastle upon Tyne, United Kingdom; ^3^Department of Nursing, Midwifery and Health, Northumbria University, Newcastle upon Tyne, United Kingdom; ^4^Faculty of Medicine and Health, University of Leeds, Leeds, United Kingdom; ^5^Paediatric Anesthesia, University Hospitals Plymouth National Health Service Trust, Plymouth, United Kingdom; ^6^Faculty of Health: Medicine, Dentistry and Human Sciences, School of Nursing and Midwifery, University of Plymouth, Plymouth, United Kingdom; ^7^Department of Nursing, Hunan Children's Hospital, Changsha, China

**Keywords:** COVID-19, parents, confidence, anxiety, health behavior, children, social isolation, impact

## Abstract

**Background:** Parents' health-seeking behaviors has changed during the COVID-19 pandemic. Providing parents with guidance in decision making might improve their confidence to seek timely advice when a child becomes ill. The aim of this study was to evaluate the “How to recognize if your child is seriously ill” leaflet on parents' confidence, health-seeking behaviors, and usefulness during the COVID-19 lockdown.

**Method:** A nine-item survey, codesigned with parent advisors, was used to measure confidence and health-seeking behavior. Social media was used for data collection in a 6-week period (April–June 2020) during COVID-19 lockdown in the United Kingdom. Categorical data were analyzed as frequencies, and inductive content analysis was performed with the qualitative data.

**Results:** In total, 171 parents responded. Most parents (*n* = 160, 93.6%) found the leaflet helpful. The leaflet increased the confidence among 116 parents (67.8%) to recognize if their child is ill, and 156 (91.2%) parents had a better understanding of when and where to seek help. Thirty-three (19.2%) parents used the leaflet, while their child was unwell during COVID-19 lockdown, and in 14 (42%) cases, the leaflet resulted in changing health-seeking behavior for that episode. Twelve of these parents decided to seek medical consultation when they had not planned to before. Content analysis revealed three categories. (1) Knowledge—parents found the leaflet an objective source to validate their concerns. (2) Usability—parents reported that the leaflet was clearly designed. (3) Decision aid—parents commented that the leaflet provided clarification around recognition of serious symptoms and when and where to seek appropriate care.

**Conclusions:** Our leaflet provided parents with guidance on decision making and risk assessment of ill children during COVID-19 lockdown. Parents found it helpful; it increased their confidence and positively changed their health-seeking behaviors. Providing parents with targeted information to recognize serious illness in children at home could potentially foster self-care and safely maintain a reduction in pediatric emergency attendances for self-limiting illnesses.

## Introduction

The COVID-19 pandemic has been marked by the sense of fear and anxiety experienced by both children and families (1, 2). The difference between the COVID-19 pandemic compared to other epidemics is the mandated social isolation and distancing measures. Closure of schools and nurseries has had a huge impact on families with children (3).

Pediatric emergency departments experienced a significant drop in attendances during the 1st months of lockdown (4). Rates of presentation have fallen by 70% since the start of the pandemic, and a report from Italy documented an increase in delayed presentations resulting in avoidable harms and increased pediatric intensive care admissions (5). Parents of unwell children might delay contacting healthcare services fearing nosocomial infection, being isolated from their child, or not wanting to burden the hospital during the pandemic. There were concerns from pediatricians that unwell children were more likely to be affected by non-COVID-19-related disease and collateral damage from delayed presentation could be significant. However, a recent study in the United Kingdom reported that delayed presentations at emergency departments were rare (4).

The COVID-19 pandemic has challenged hospitals in preparing effective response systems (6). Providing and disseminating timely, clear, and reliable information for parents has been challenging. The UK National Health Service (NHS) provided guidance for COVID-19 patients in primary care and family doctor services (7). For children, COVID-19 information has been described and explained by Alberca et al. (8), and children's hospitals created webpages to inform children and families (9).

Prior to the COVID-19 pandemic, our team of parents and healthcare professionals developed a parent decision-making and risk assessment leaflet (10) for parents to identify early deterioration of a seriously ill child, including *parental concern* as a trigger to escalate care (11). Due to the decrease in pediatric emergency department (PED) attendances at the early stages of the COVID-19 pandemic, we published our leaflet on the hospital website in February 2020 to support parents in the community. We considered that this intervention would give parents the confidence to access timely healthcare services. To our knowledge, there is limited evidence assessing the impact of advice around healthcare-seeking behavior and parental information during the COVID-19 pandemic.

The aim of this study was to evaluate the experiences of parents using this decision-making and risk assessment leaflet for a potentially seriously ill child during COVID-19 lockdown. More specifically, we explored the confidence of parents, their health-seeking behaviors, and usefulness of the leaflet.

## Materials and Methods

An observational service evaluation design was adopted. The “Guidelines for Reporting Evaluations based on Observational Methodology” (GREOM) was used to report this study (12).

The Research and Design Service of the Newcastle Upon Tyne Hospitals NHS Foundation Trust approved this study as a service evaluation. The survey was anonymous, and confidentiality of information was assured.

### Parent Leaflet

The parent decision-making and risk assessment leaflet “How to recognize if your child is seriously ill” was developed with parents for parents (Electronic Supplementary Material 1). The content was designed using the NICE “Fever in under 5s: assessment and initial management” (13), pediatric sepsis guidelines, sepsis leaflets for parents (14–16), and studies reporting signs and symptoms of children presenting to pediatric emergency departments (17, 18). Based on the available evidence, the content of the risk assessment symptoms refers to preschool and primary school children, generally below 12 years of age. Some symptoms have been included when these are specifically related to children below 1 year or 1 month of age.

The leaflet includes two main sections. The first section explains how to use the leaflet and provides space to write down the details of the child and parental concerns. Additional practical tips and medication use are included. The second section is divided into green, amber, and red with guidance for decision making of where and how to seek health advice. The risk assessment includes six systems: (1) temperature; (2) breathing; (3) skin, lips, and tongue; (4), eating and drinking, (5) toilet/nappies; and (6) activity and body.

Ten parents from a local parent support group reviewed the content of the leaflet and compared it with another leaflet (19) published by the Royal College of Pediatrics and Child Health (RCPCH) during the early stages of the COVID-19 pandemic. Overall, there was consensus that our parent decision-making and risk assessment leaflet was preferred as it included parental concern, sufficiently detailed information, and options to write down observations.

The distribution of the parent leaflet was implemented without manipulation and could be used in the everyday context during the COVID-19 lockdown. The evaluation was conducted via an online survey with a minimum number of questions designed to be completed in < 5 min to reduce the burden to respondents during COVID-19 lockdown and was considered a low-intensity intervention in the context of the observational methods.

### Participants and Recruitment

Participants were parents, carers, or guardians of children in the North East of England during the COVID-19 lockdown. The population in the North East of England is around 320,000, and the last census in 2011 documented that 97% of the population described themselves as white British (20).

We anticipated a sample size of around 100 parents based on previous work evaluating a parent sepsis leaflet in the South West of England (21). The sample size depended on the voluntary engagement of parents in the community and the promotion of the leaflet. Inclusion criteria were parents, caretakers, or guardians with children who downloaded the parent leaflet and the attached invitation with a website link to complete a short online survey.

The method of recruitment was via social media. The leaflet and an invitation to participate was distributed via Twitter, Facebook, and the website of our hospital. Local schools, social services, and COVID-19 support groups informed parents via their chat groups, tweets, and messages on Facebook containing a link to the leaflet and online survey.

The lockdown in the United Kingdom started on March 12, 2020 and was eased on June 15, 2020 allowing the opening of non-essential shops. The recruitment time via social media was 6 weeks, from April 29, 2020 to the first ease of the COVID-19 lockdown on June 15, 2020. We acknowledged that recruitment via social media might include respondents from outside North East of England. Therefore, we included all respondents in the United Kingdom but excluded those outside the United Kingdom.

### Outcomes and Data Collection

The self-administered online survey was designed to address parent's confidence, health-seeking behavior, and general experience of the leaflet. The survey was developed together with our local parent advisory group. Ten parents reviewed the draft of the survey, and minor suggestions were provided to improve the clarity of the questions and answer option scales. Parents agreed that the final 10-item survey was acceptable to complete in the current COVID-19 climate.

The final survey included eight closed questions and two optional open-ended questions (Electronic Supplementary Material 2). The first three questions were demographic questions followed by questions measuring health-seeking behavior, experience of the leaflet, and confidence. Two open-ended questions were included to provide parents the option of sharing their experiences and opinions.

### Data Analysis

The quantitative analyses were performed using IBM-SPSS version 25.0 (IBM, New York, NY, USA). Categorical data are presented as frequency in percentages. The responses of the two open-ended questions were analyzed by inductive content analysis (22). This included open coding of the narratives and grouping into subcategories. Abstraction was further enhanced by generating categories described by content-characteristic words.

## Results

During the 6-week data collection, 171 parents responded to the online survey. Of the 170 respondents who completed the postcode question, 148 (86.5%) were living in the North East of England, and 22 (13.5%) were living in the rest of the United Kingdom. Half of the parents (*n* = 87; 50.9%) reported having two children in their household followed by 46 (26.9%) parents having one child (Table 1). Most of the parents (*n* = 159, 93%) were white British reflecting the population ethnicity in the North East of England (Table 1).

**Table 1:** Characteristics of participants (*n* = 171).

**Parents**	**n (%)**
**Number of children in household**	
1	46 (26.9)
2	87 (50.9)
3	33 (19.3)
4	5 (2.9)
**Ethnicity**	
White British	159 (93.0)
White other	4 (2.3)
White and Asian	2 (1.2)
White and Black African	2 (1.2)
White Irish	1 (0.6)
Indian	1 (0.6)
Black or Black British	1 (0.6)
Other mixed	1 (0.6)
**Region in UK**	
North East of England	148 (86.5)
Rest of UK	22 (13.5)

In terms of health-seeking behaviors and first point of contact when their child is unwell, 87 (50.9%) of the parents reported that they usually visit and seek advice from their family doctor. A further 59 (34.5%) parents reported that they first would call NHS 111, a free non-emergency medical helpline. Only 14 (8.2%) parents reported that they would visit the emergency department (Table 2).

**Table 2:** Experiences of parent information leaflet (*n* = 171).

**Parents**	**n (%)**
**What would you normally do if you were worried about your ill child?**	
A&E	14 (8.2)
NHS 111	59 (34.5)
Home	2 (1.2)
GP	87 (50.9)
Walk-in center	9 (5.2)
**How helpful did you find this leaflet?**	
Very helpful/somewhat helpful	160 (93.6)
Neutral	10 (5.8)
Not helpful at all/a little unhelpful	1 (0.6)
**After reading this leaflet, how do you feel about recognizing if your child is seriously ill?**	
More confident	116 (67.8)
The same	55 (32.2)
Less confident	0
**After reading this leaflet, do you have a better understanding of when and where to seek the right healthcare for your ill child?**	
No better	15 (8.8)
A bit better	90 (52.6)
A lot better	66 (38.6)
**If you are using this because your child is unwell, was this decision different to what you thought before?**	
Yes, seek medical attention	12 (7.0)
Yes, did not seek medical attention	2 (1.2)
The same	19 (11.1)
Not applicable	138 (80.7)

*A&E, Emergency department; GP, general practitioner (family doctor)*.

Overall, most parents perceived the leaflet as being helpful (93.6%). Of these, 89 (52%) parents found the leaflet very helpful, and 71 (41.5%) as somewhat helpful. The leaflet increased the confidence of parents (*n* = 116, 67.8%) in recognizing if their child is seriously ill (Table 2). Furthermore, 156 (91.2%) parents reported that they had a better understanding of when and where to seek help. There were 33 (19.3%) parents who used the leaflet while their child was unwell, and in 14 (42%) cases, the leaflet resulted in changing the health-seeking behavior for that episode. Of these, 12 parents chose to take their child to seek medical attention where they had not planned to before, and 12 of these 14 parents reported an increased in confidence in recognizing if their child was unwell. In the group of parents where their health-seeking behavior was not changed (n = 19), only 10 parents reported an increase in confidence.

Content analysis of the free text responses revealed three categories: Knowledge, Usability, and Decision aid (Table 3). The category Knowledge included three subcategories where parents commented on the leaflet as being an objective source to validate their concerns. Parents wrote that the leaflet was an *essential resource* while staying at home. The leaflet was valued by parents because of the *age specificity* of some of its information. This helped parents in their *medical assessment* when their child was becoming sick. The category Usability included the subcategories *linguistic clarity* and *visual clarity* where parents reported that the leaflet was clearly designed including the traffic light color coding system on when and how to seek medical attention. The category Decision aid had two subcategories. The subcategory *response calibration* was related to the systematic breakdown of symptoms that provided clarification about what to observe, when to make the decision to seek further advice, and who to contact. Some parents reported that the leaflet gave them more *confidence* in their decision making.

**Table 3:** Parent's experiences of leaflet.

**Categories**	**Subcategories**	**Narratives**
Knowledge	Essential resource	This sort of thing should be given to new parents on discharge from NICU or postnatal wards, too. I feel especially at the moment (R65)
		Concise and something that can stay in inside of the cupboard where medicines are kept (R132)
	Age specificity	I liked that there was more specific advice for newborns and under ones as often information is not at all age specific (R43)
		The exact temperatures for various ages are useful (R13)
		My child is under 1, and I now feel I like more of the signs to look out for in his age group (R74)
	Medical assessment	It makes it simpler to assess symptoms as serious or less serious. It brings lots of information into one place that is easy to use. (R72)
		It has lots of signs to look out for which some parents might not know what to look out for (R2)
		It helps focus your mind on the signs that potentially are the more serious ones to look for and separate those out clearly to help you make more of an informed decision about what person/service you should contact or what care might be suitable (R65)
Usability	Linguistic clarity	I found the leaflet very informative and well-structured. I like how it has clear points […] and think it is really easy to follow (R66)
		Very informative leaflet with clear information (R165)
	Visual clarity	The traffic light system is excellent and simple to understand (R65)
		Good to have a checklist to refer to especially when worried about your child (R135)
Decision aid	Response calibration	It helped clarify the difference between seeking the GP's advice and going to hospital (R80)
		It is good to see it broken down in terms of symptoms and response need (R119)
	Confidence	There were times when my children were younger when in hindsight I should have taken them in to hospital. It was have bolstered my confidence that I was not “making a fuss” if I would read this (R119)
		Much more information than you can usually get from leaflets. It would make me more confident and less likely to start googling, which usually makes things even more confusing! (R43)

The same categories were used in analyzing the free-text responses related to suggestions for improvements (Table 4). In the Knowledge category, the *risk guidance* was deemed to be unclear by some parents, and they cautioned over the ambiguity of symptoms to seek help in the amber category of the leaflet. The category Usability included comments on the *linguistic* and *visual clarity*. Two parents commented that the leaflet could be written in easier language as the leaflet could be challenging for parents with low literacy skills. Another parent suggested using bullet points rather than tick boxes before every symptom. In the Decision aid category, parents suggested that the leaflet could increase *confidence* by emphasizing parental concern (or gut instinct) more prominently. Three parents highlighted that the leaflet does not cover specific emergencies such as anaphylaxis, diabetic ketoacidosis, febrile convulsions, or meningitis.

**Table 4:** Suggestions provided by parents.

**Categories**	**Subcategories**	**Narratives**
Knowledge	Risk Guidance	The only question I have is in the amber bit. Child not quite right; this would fit for both children every time they are ill, but they do not always need healthcare (R139)
		I was only a little confused by amber—if they show just 1 of these symptoms, is it amber, or is it several? The red seemed clearer in that all those symptoms would alarm me, and it is reassuring that I am right to be alarmed and just get hold of emergency services (R172)
		High-risk factors are obvious and clear. The amber ones could be taken out of context. What if baby just has one of those listed, is that enough to be worried? Some of those symptoms could relate to something else and not cause a great deal of concern (R112)
Usability	Linguistic clarity	Some of the language could be simpler. Especially in the last page for management of fever. Do not give the child a shower or cold cloths as it will spike the temperature. Do not lower the temperature too quickly. The language on alternating paracetamol and ibuprofen could be clearer, too (R128)
		Although I can understand, it there is a lot of writing, which pay but [may put] some people off from looking at it especially those with limited English or poor literacy skills (R26)
	Visual clarity	The squares in the red and amber make it a tick list—like you are counting symptoms—that is not a message, is it? It is, if your child has any of these symptoms. It should be bullet points (R47)
Decision-aid	Confidence	The “less interested” statement is really vague. It would be better to have something about parents' instinct that something is wrong (R102)
	Disease-specific advice	Information about febrile seizures would be useful to include, like what to do if they have a seizure (R128)

## Discussion

This project aimed to explore the experiences of parents using an information leaflet to help them recognize serious illness in their children and respond appropriately during the COVID-19 lockdown. The leaflet provided parents with detailed information to support decision making and seek timely medical advice. Our leaflet was coproduced with local parents via an iterative process. Information material that is coproduced and evaluated by target stakeholders has been shown to be more effective in improving knowledge and health-seeking behaviors (23).

Parents of unwell children are in a position of vulnerability and are aware of being perceived as worriers by healthcare professionals and their friends/family (24, 25). Parents fear hospital attendance with its perceived risks, while social distancing has removed normal access to formal and informal social support structures. Febrile illnesses increase parental anxiety (26), especially when other symptoms are already present (25). This was clearly heightened during the COVID-19 pandemic (27), especially with fever being a central tenet of the case definition. Parents desire a clear source of information that they can independently access to empower themselves, validate their concerns, and reassure them that they have taken the appropriate course of action when caring for their febrile child (25). Our leaflet provided this through detailed and comprehensive information.

Baseline knowledge is vital, and gaps in parents' knowledge have been identified; most parents cannot identify the temperature at which a child is said to be febrile (25). This has resonance with our findings as many respondents commented positively on our inclusion of age-specific temperature thresholds. Supplying information on management is crucial to guide appropriate and timely use of healthcare services for parents and avoid unnecessary consultations (28). Importantly, comprehensive multitopic information on febrile illness is more effective at reducing parental anxiety and increasing confidence than single differentials of fevers (26). This is because children often present with a constellation of symptoms alongside a fever.

Most parents in our study would see their GP first. This health-seeking behavior is well-established with parents in high-income countries (25). Often parents consult printed or electronic reference material before deciding what course of action to take (26). There is limited evidence on parents' health-seeking behavior during COVID-19. The NHS has noted a one-third drop in emergency department attendances (29) and GP appointments (30) coupled with a 50-time rise in online triage information being accessed (31) during the current outbreak. This indicates many more people are seeking authoritative reference material prior to attending and suggests there is an opportunity to influence primary care and PED attendance.

Several limitations need to be addressed. We used an online web-based survey and recruitment via social media both of which could have resulted in selection bias. Parents who have limited access to social media platforms, the Internet, computers, or smartphones may not have had the opportunity to be included or been able to access the leaflet. This may be a particular issue in socially deprived areas or in middle- and low-income countries without ready Internet access. Newcastle-upon-Tyne has large socially deprived areas, and we worked together with local charities such as the Newcastle West End Schools Trust and Newcastle local authority and agencies affiliated with the West Partnership who kindly supported the project in printing and distributing the parent leaflet to ensure wide access. We also received support from community groups, schools, and health visitors who were willing to advertise the parent leaflet on their websites. We also acknowledge the limitation of the short survey. The survey was designed to generate a general understanding of the parents' experiences, confidence, and health-seeking behaviors and was intentionally kept short to reduce the burden of parents to complete the survey. Validated instruments exist, such as the 34-item Family Empowerment Scale (32) and the 15-item Karitane Parenting Confidence Scale (33), but are significantly longer to administer.

In conclusion, the majority of parents found our parent information leaflet on decision making and risk assessment in ill children helpful and improved their confidence. The leaflet may have changed health-seeking behavior during the COVID-19 social isolation period and contributed to patents' better understanding of when and where to seek medical attention if their child becomes unwell.

The silver lining to the COVID-19 pandemic has been the reduction in PED attendances. Providing parents with targeted information when a child becomes seriously ill at home could potentially foster self-care and safely maintain this reduction of PED attendances for self-limiting illnesses. Our study shows parents welcoming this information and are actively seeking trusted sources of healthcare information and acting on them. The onus is on healthcare practitioners and institutions to understand this need and deliver quality healthcare support and education widely. Now, we need further research to understand the effectiveness of our parent information leaflet during the “new normal” including children's (long-term) health outcomes, particularly focusing on the most disadvantaged sectors and their access to health information.

## Data Availability Statement

The raw data supporting the conclusions of this article will be made available by the authors, without undue reservation.

## Ethics Statement

The Research and Design Service of the Newcastle upon Tyne Hospitals NHS Foundation Trust approved this study as a service evaluation.

## Author Contributions

EL, AB, KD, and JML developed the intervention. EL, AB, KD, AK, MNC, MCC, and JML initiated the evaluation and contributed to the design of the study. EL, AB, KD, and RM contributed to the data collection. EL, RM, MCC, and JML contributed to the data analysis and interpretation. EL and JML drafted the first manuscript. All authors contributed to manuscript revisions, approved the final version of the manuscript, and agreed to be accountable for the content of the work.

## Conflict of Interest

The authors declare that the research was conducted in the absence of any commercial or financial relationships that could be construed as a potential conflict of interest.
