# Supplementary material for: “How to Recognize if Your Child Is Seriously Ill” During COVID-19 Lockdown: An Evaluation of Parents' Confidence and Health-Seeking Behaviors
Source: Front Pediatr. 2020 Nov 17;8:580323. doi: 10.3389/fped.2020.580323 (PMC7707121; doi:10.3389/fped.2020.580323)
Supplement: Supplementary file 1 [file Table_1.DOCX]

Supplementary Material 1

**‘How to recognize if your child is seriously ill’ during COVID-19 lockdown: A service evaluation of parents’ self-confidence and health-seeking behaviors**

Emma LIM, Alexandra BATTERSBY, Kerry DOCKERTY, Ravi MISTRY, Aaron KOSHY, Michelle CHOPRA, Matthew CAREY,

Jos M. LATOUR

## Supplementary File 1: The parent decision-making and risk assessment leaflet ‘How to recognize if your child is seriously ill’
