# Supplementary material for: “How to Recognize if Your Child Is Seriously Ill” During COVID-19 Lockdown: An Evaluation of Parents' Confidence and Health-Seeking Behaviors
Source: Front Pediatr. 2020 Nov 17;8:580323. doi: 10.3389/fped.2020.580323 (PMC7707121; doi:10.3389/fped.2020.580323)
Supplement: Supplementary file 2 [file Table_2.DOCX]

Supplementary Material 2; Online Survey

**‘How to recognize if your child is seriously ill’ during COVID-19 lockdown: A service evaluation of parents’ self-confidence and health-seeking behaviors**

Emma LIM, Ravi D. MISTRY, Alexandra BATTERSBY, Kerry DOCKERTY, Aaron KOSHY, Michelle CHOPRA, Matthew CAREY, Jos M. LATOUR

| **Question** | **Answer option** |
| --- | --- |
| How many children do you have? | Number |
| What ethnic group would you put yourself in? | White British; White Irish; Other White; White and Black Caribbean; White and Black African; White and Asian; Other mixed Group; Asian or Asian British; Indian; Pakistani; Bangladeshi; Other Asian Ethnic Group; Black or Black British; Caribbean; African; Other Black Ethnic Group; Chinese, Rather not say; Other |
| What is the first part of your postcode?  For example, NE15 or NE3 (as appropriate) | Open space |
| What would you normally do if you were worried about your ill child? | - Go to A&E - Phone 111 - Stay at home - Go to the GP - Go to the walk-in centre |
| How helpful did you find this leaflet? | 1. Not helpful at all 2. Somewhat helpful 3. Neutral 4. A little unhelpful 5. Very helpful |
| Optional: Please tell us more | Free text |
| After reading this leaflet, how do you feel about recognising if your child is seriously ill? | - Less confident - The same - More confident |
| Optional: Please tell us more | Free text |
| After reading this leaflet, do you have a better understanding of when and where to seek the right healthcare for your ill child? | - No better - A bit better - A lot better |
| If you are using this because your child is unwell: was this decision different to what you thought before? | - Yes, I didn’t seek medical attention and I would have - Yes, I went to seek medical attention and would have stayed at home - No, it was the same - Not applicable |
